# Leukocyte Mitochondrial DNA Alteration in Systemic Lupus Erythematosus and Its Relevance to the Susceptibility to Lupus Nephritis

**DOI:** 10.3390/ijms13078853

**Published:** 2012-07-16

**Authors:** Hui-Ting Lee, Chen-Sung Lin, Wei-Sheng Chen, Hsien-Tzung Liao, Chang-Youh Tsai, Yau-Huei Wei

**Affiliations:** 1 Institute of Clinical Medicine, National Yang-Ming University, Taipei 112, Taiwan; E-Mails: htlee1228@gmail.com (H.-T.L.); doc2765c@ms59.hinet.net (C.-S.L.); curtis3625@yahoo.com.tw (W.-S.C.); 2 Faculty of Medicine, National Yang-Ming University, Taipei 112, Taiwan; 3 Division of Allergy, Immunology and Rheumatology, Department of Internal Medicine, Wan-Fang Hospital, Taipei Medical University, Taipei 116, Taiwan; E-Mail: darryliao@yahoo.com.tw; 4 Division of Thoracic Surgery, Department of Surgery, Taipei Hospital, Department of Health, Executive Yuan, New Taipei City 242, Taiwan; 5 Division of Allergy, Immunology and Rheumatology, Department of Internal Medicine, Taipei Veterans General Hospital, Taipei 112, Taiwan; 6 Institute of Biochemistry and Molecular Biology, National Yang-Ming University, Taipei 112, Taiwan; 7 Department of Medicine, Mackay Medical College, New Taipei City 252, Taiwan

**Keywords:** systemic lupus erythematosus, mitochondrial DNA, copy number, D310 sequence variation, heteroplasmy

## Abstract

The role of mitochondrial DNA (mtDNA) alterations in the pathophysiology of systemic lupus erythematosus (SLE) remains unclear. We investigated sequence variations in the D310 region and copy number change of mtDNA in 85 SLE patients and 45 normal subjects. Leukocyte DNA and RNA were extracted from leukocytes of the peripheral venous blood. The D310 sequence variations and copy number of mtDNA, and mRNA expression levels of mtDNA-encoded genes in leukocytes were determined by quantitative real-time polymerase chain reaction (Q-PCR) and PCR-based direct sequencing, respectively. We found that leukocyte mtDNA in SLE patients exhibited higher frequency of D310 heteroplasmy (69.4% *vs.* 48.9%, *p* = 0.022) and more D310 variants (2.2 *vs.* 1.7, *p* = 0.014) than those found in controls. Among normal controls and patients with low, medium or high SLE disease activity index (SLEDAI), an ever-increasing frequency of D310 heteroplasmy was observed (*p* = 0.021). Leukocyte mtDNA copy number tended to be low in patients of high SLEDAI group (*p* = 0.068), especially in those harboring mtDNA with D310 heteroplasmy (*p* = 0.020). Moreover, the mtDNA copy number was positively correlated with the mRNA level of mtDNA-encoded ND1 (NADH dehydrogenase subunit 1) (*p* = 0.041) and ATPase 6 (ATP synthase subunit 6) (*p* = 0.030) genes. Patients with more D310 variants were more susceptible to lupus nephritis (*p* = 0.035). Taken together, our findings suggest that decrease in the mtDNA copy number and increase in D310 heteroplasmy of mtDNA are related to the development and progression of SLE, and that the patients harboring more D310 variants of mtDNA are more susceptible to lupus nephritis.

## 1. Introduction

Systemic lupus erythematosus (SLE) is a prototype of autoimmune disease characterized by the dysfunction of immunocompetent cells with the production of protean pathogenic auto-antibodies that lead to multiple arrays of major organ injuries [1]. Although the pathogenesis of SLE has been attributed to several environmental or genetic factors, the detailed mechanisms remain obscure [2]. Previous studies have revealed that mitochondrial dysfunction may participate in the pathogenesis of SLE through its implication in causing insufficient ATP production for cell survival and triggering apoptosis and autophagy for cell death [3–6]. Furthermore, recent studies showed that genetic alterations in mitochondrial DNA (mtDNA) were associated with the susceptibility to SLE [7,8], and an increase of mtDNA content in serum was related to a poor outcome of SLE [9]. Thus, a delineation of the role of mtDNA alterations in SLE may be of great clinical relevance.

Human mtDNA is a double-stranded and circular DNA that contains approximately 16,569 nucleotide base pairs (bp), including the coding and non-coding regions (refer to a human mitochondrial genome database) [10,11]. The coding region encodes 13 polypeptides relevant to electron transport as well as a set of 22 tRNAs and 2 rRNAs required for protein synthesis in mitochondria. All of the 13 polypeptides encoded by mtDNA are essential for the assembly of respiratory enzyme complexes (Complexes I, II, III, and IV for electron transport; and Complex V for oxidative phosphorylation), including 7 (ND1, ND2, ND3, ND4, ND4L, ND5, and ND6; ND = NADH dehydrogenase) in Complex I, 1 (cytochrome *b*) in Complex III, 3 (COX I, COX II and COX III; COX = cytochrome *c* oxidase) in Complex IV, and 2 (ATPase 6 and ATPase 8; ATPase = ATP synthase) in Complex V. Except for the 13 polypeptides, all the other polypeptides composing respiratory chain are encoded by nuclear DNA (nDNA) [10,12]. Each human cell contains several hundreds to one thousand of mitochondria, and each mitochondrion harbors 2 to 10 copies of mtDNA. The mtDNA copy number in a human tissue is dynamic and may vary widely with cell type and the physiological condition [13]. Different from the heterozygotic nature of nDNA, mtDNA is transmitted exclusively through the maternal lineage [14,15]. The mtDNA molecules in the post-mitotic tissues of an individual are assumed to be identical immediately after birth and such a condition is termed homoplasmy. Any damage to mtDNA may disrupt the homoplasmy and result in the coexistence of the wild type and mutated mtDNA molecules, which is termed heteroplasmy [14,15].

Due to the lack of introns and histone protection, insufficient DNA repair system and the location near the inner membrane of mitochondria with a milieu of high concentration of reactive oxygen species (ROS), mtDNA is far more susceptible to oxidative damage as compared to nDNA [16–18]. Although the damages can occur anywhere throughout the entire mtDNA, alterations are frequently found in the non-coding region, the displacement loop (D-loop), especially in the D310 region [19]. D-loop is the regulatory region for replication and transcription of mtDNA and is located between nucleotide position (np) 16024 and np 576 with 1.1 kb in size [10,12]. Between np 303 and np 316 of the D-loop (revised Cambridge Reference Sequences, L strand sequences; rCRS), there is a poly-cytidine (C) tract with a thymidine (T) interrupted at np 310 (-C_303_CCCCCCT_310_CCCCCC_316_- = C_7_TC_6_), and a constant stretch of 6 C after T_310_. However, the number of C before T is highly variable with a range from 6 to 12 (C_6_, C_7_, C_8_, C_9_, C_10_, C_11_, C_12_), and 7 or 8 (C_7_ or C_8_, wild-type) being the most common ones [10]. These variations with a T shifting over np 310 (originated from a deletion or insertion) in mtDNA are referred to as D310 polymorphism or D310 sequence variations. Sequence variations in the D310 region have been identified in a variety of human diseases, including cancers, degenerative diseases and some hereditary diseases [20–24].

In this study, we examined the differences in the copy number and D310 sequence variations of mtDNA in leukocytes of SLE patients and normal subjects. Furthermore, we investigated whether these differences are associated with clinical manifestations of SLE.

## 2. Results and Discussion

### 2.1. Demographic Data of the SLE Patients and Controls

**Figure 1:**
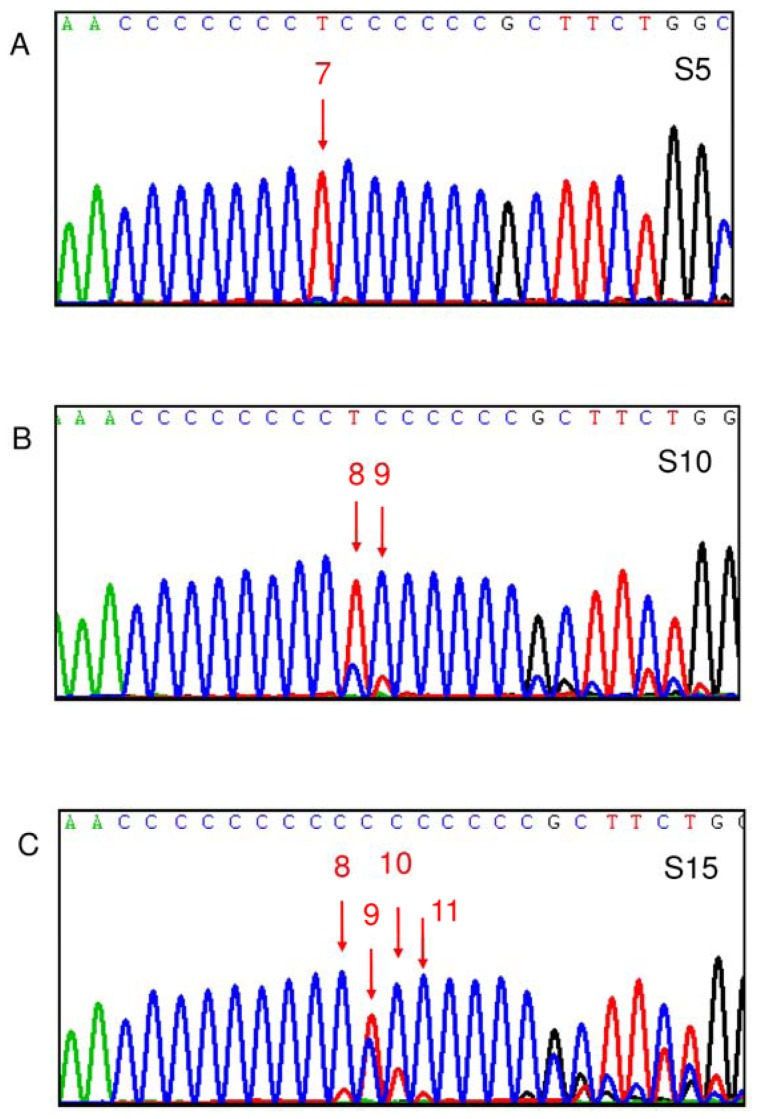
Representative cases to demonstrate the D310 sequence variations. T (thymine) is shown in red, A (adenine) in green, C (cytosine) in blue and G (guanine) in black during sequencing. The Arabic number above the red arrow denotes the number of C before the indicated T peak. (**A**) In patient S5, leukocyte mtDNA harbored 1 kind of D310 variant, C_7_TC_6_. As a result, S5 was classified as D310 homoplasmy with C_7_TC_6_ being the dominant variant; (**B**) In patient S10, leukocyte mtDNA harbored 2 kinds of D310 variants, C_8_TC_6_ and C_9_TC_6_ in order, with C_8_TC_6_ being the dominant one. As a result, S10 was classified as D310 heteroplasmy with C_8_TC_6_ as the dominant variant; (**C**) In patient S15, leukocyte mtDNA harbored 4 kinds of D310 variants, C_9_TC_6_, C_10_TC_6_, C_8_TC_6_ and C_11_TC_6_ in order, with C_9_TC_6_ being the dominant one. As a result, S15 was classified as D310 heteroplasmy with C_9_TC_6_ as the dominant variant. Clinically, patient S15 developed lupus nephritis.

The demographic data were compared between 85 SLE patients and 45 age- and sex-matched normal individuals. As shown in Table 1, the SLEDAI scores of the 85 patients ranged from 2 to 28 with a median value of 8. Neuropsychiatric disturbance, lupus nephritis, skin rash, alopecia, oral ulcer and decreased serum complements were the predominant clinical manifestations. Regarding the D310 sequence variations (Figure 1), the percentage of SLE patients harboring C_9_TC_6_ as the dominant variant in the D310 region is slightly higher than that of controls (24.7% *vs.* 11.1%, *p* = 0.079, Chi-square test). Interestingly, SLE patients had a higher frequency of D310 heteroplasmy and more D310 variants than did controls (69.4% *vs.* 48.9%, *p* = 0.022, Chi-square test and 2.2 *vs.* 1.7, *p* = 0.014, Student *t* test). The mtDNA copy number in controls and in SLE patients were not significantly different (0.193 ± 0.065 *vs.* 0.214 ± 0.113, *p* = 0.169, Student *t* test), although mildly elevated in SLE patients. The mRNA expression levels of mtDNA-encoded ND1 and ATPase 6 genes in the controls (*n* = 26) were significantly higher than those of SLE patients (*n* = 44) (2.845 ± 2.000 *vs.* 1.625 ± 1.029, *p* = 0.001, ND1; 1.538 ± 1.108 *vs.* 0.928 ± 0.526, *p* = 0.003, ATPase 6; Student *t* test).

### 2.2. Correlation between SLEDAI and Leukocyte mtDNA in SLE Patients

**Figure 2:**
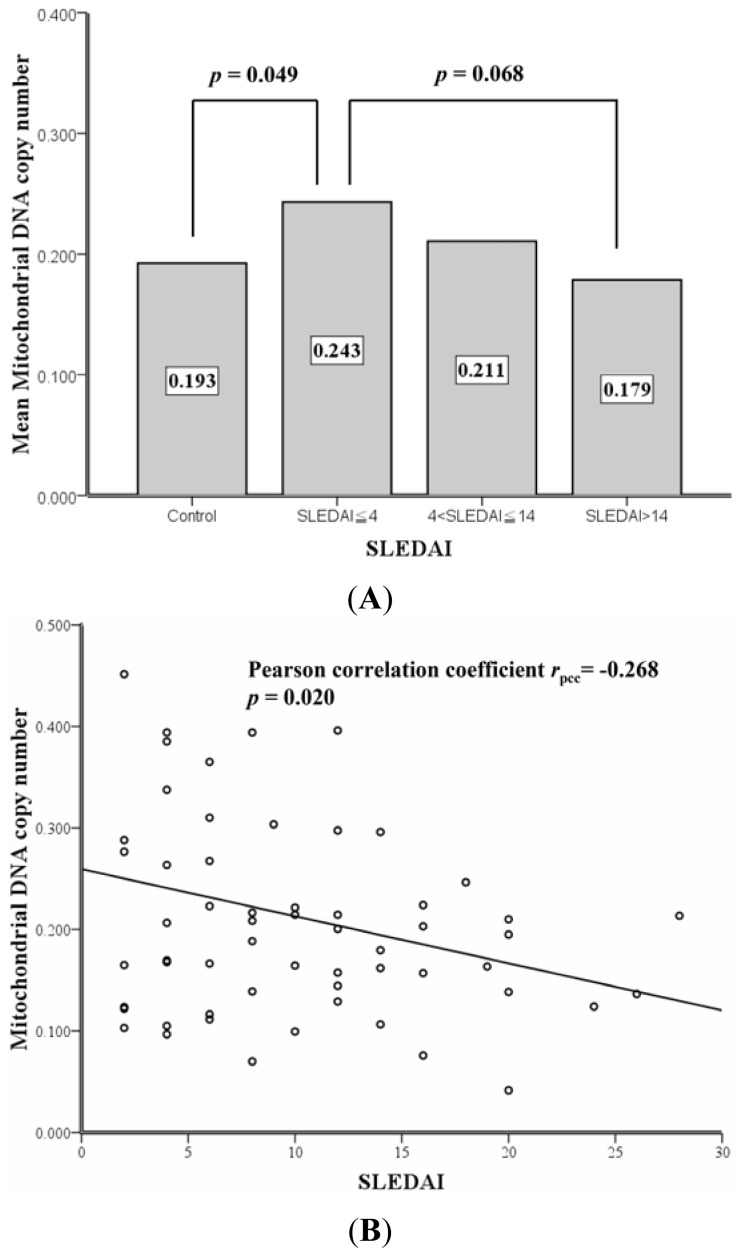

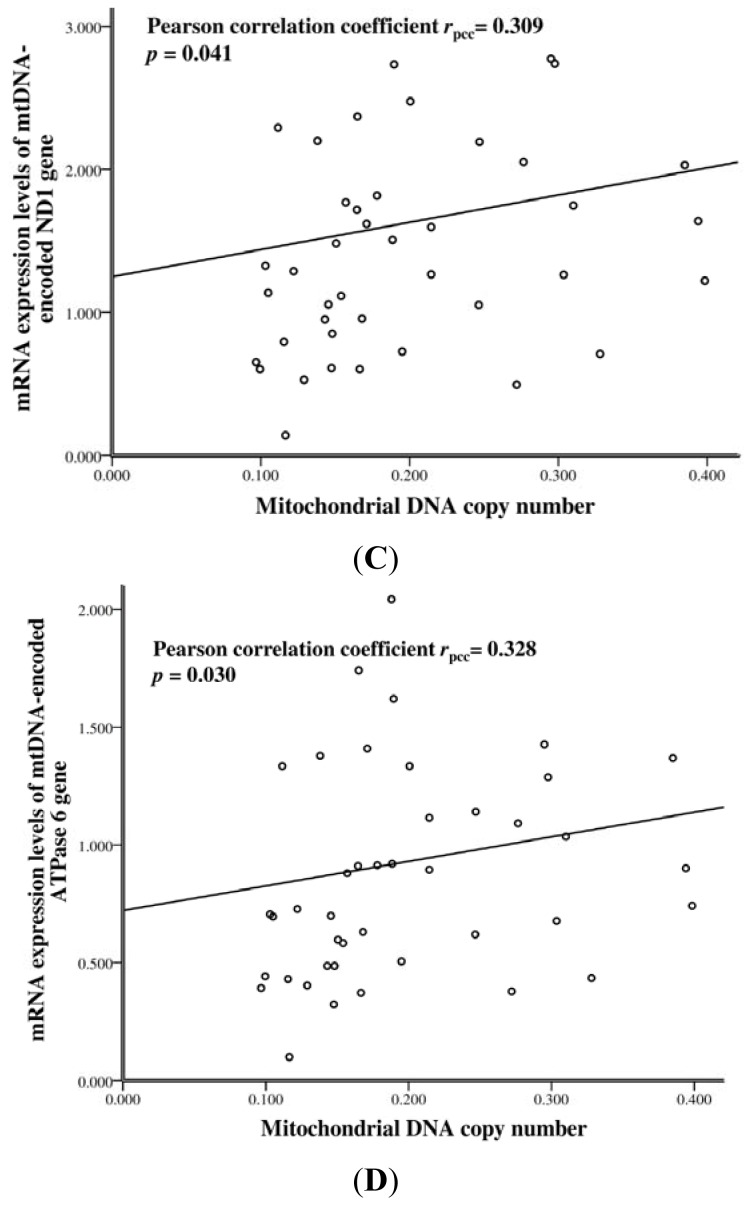
(**A**) The distribution of mean mtDNA copy number in the leukocytes of normal controls and SLE patients with low, medium and high SLE Disease Activity Index (DAI) scores. The mean mtDNA copy number in the leukocytes of the group with low SLEDAI score (0.243 ± 0.147) was higher than that of controls (0.193 ± 0.065) (*p* = 0.049) and the group with a high SLEDAI score (0.179 ± 0.077, *p* = 0.068); (**B**) In SLE patients (*n* = 59) with D310 heteroplasmy in mtDNA of the leukocytes, we found a negative correlation between the mtDNA copy number and SLEDAI score (*p* = 0.020, Pearson’s correlation coefficient γ_pcc_ = −0.268); (**C**) Among the 44 analyzed SLE patients, a positive correlation was found between mtDNA copy numbers and the mRNA expression levels of mtDNA-encoded ND1 gene; (**D**) Among the 44 analyzed SLE patients, a positive correlation was found between mtDNA copy numbers and the mRNA expression levels of mtDNA-encoded ATPase 6 gene.

As shown in Table 2, SLE patients were divided into 3 groups based on the 25 percentile and 75 percentile of SLEDAI scores, including SLEDAI ≤ 4 (low SLEDAI group, ≤25%, *n* = 26), 4 < SLEDAI ≤ 14 (medium SLEDAI group, 26%~75%, *n* = 42) and SLEDAI > 14 (high SLEDAI group, >75%, *n* = 17). Among controls and SLE patients with low, medium and high SLEDAI scores, the frequency of D310 heteroplasmy was steadily increased (48.9%, 65.4%, 69.0% & 76.5%, *p* = 0.021, Chi-square test for trend, Table 2). With regard to the mtDNA copy number in leukocytes, there were no significant differences between controls and SLE patients with low, medium and high SLEDAI scores (*p* = 0.119, ANOVA). However, the mean mtDNA copy number of patients with low SLEDAI score was higher than that of controls (0.243 *vs.* 0.193, *p* = 0.049, Student *t* test) and patients with high SLEDAI scores (0.243 *vs.* 0.179, *p* = 0.068, Student *t* test; Table 2 and Figure 2A). An ever-decreasing mtDNA copy numbers in the leukocytes of patients from low, medium and high SLEDAI groups (mean = 0.243, 0.211 and 0.179, Figure 2A) were observed. Such a trend of decrease was more pronounced in patients harboring D310 heteroplasmy (*n* = 59), whose mtDNA copy numbers were inversely correlated with the SLEDAI scores (*p* = 0.020, Pearson correlation coefficient *r*_pcc_= −0.268, Figure 2B).

### 2.3. The mtDNA Copy Number and mRNA Levels of mtDNA-Encoded Genes in SLE Patients

As shown in Figure 2C,D, among the 44 SLE patients, the mean mtDNA copy number of leukocytes was positively correlated with the mRNA level of mtDNA-encoded ND1 (*p* = 0.041, Pearson correlation coefficient *r*_pcc_= 0.309) and ATPase 6 (*p* = 0.030, Pearson correlation coefficient *r*_pcc_= 0.328) genes, respectively.

We demonstrated that the frequency of leukocyte D310 heteroplasmy was increased and the copy number of leukocyte mtDNA was decreased in SLE patients as their disease worsened (Table 2, Figure 2A). The decrease in leukocyte mtDNA copy number was more pronounced in SLE patients that harbored mtDNA with D310 heteroplasmy in leukocytes (Figure 2B). Furthermore, the decrease in mtDNA copy number was proportionally correlated to the decrease in the mRNA expression levels of mtDNA-encoded genes, including the ND1 that participates in the electron transport (Figure 2C) and ATPase 6 that participates in the oxidative phosphorylation (Figure 2D). In addition, pyruvate dehydrogenase (PDH) plays a pivotal role in regulating the Krebs cycle to guarantee the execution of electron transport and ATP production. Based on our unpublished data, the mean mRNA expression level of PDH in controls was much higher than in SLE patients (0.434 ± 0.497 *vs.* 0.264 ± 0.118, *p* = 0.032, Student *t* test). Moreover, the PDH mRNA expression was positively correlated with the mRNA expression of ND1 (*p <* 0.001, Pearson correlation coefficient *r*_pcc_= 0.553) and ATPase 6 (*p <* 0.001, Pearson correlation coefficient *r*_pcc_= 0.561), respectively (data not shown). As a result, the decrease in the mRNA expression levels of ND1 and ATPase 6 denotes the possibility of leukocyte mitochondrial dysfunction. Similarly, by using cDNA microarray of peripheral blood in a cohort of 26 SLE patients, Lee *et al.* [24] disclosed a decrease in the expression of 6 mtDNA-encoded genes that are responsible for ATP synthesis, including ND1 and ATPase 6 examined in this study. They concluded that the ATP synthesis is impaired in SLE patients. As we know, mitochondria play a dual role in cell survival and cell death due to their roles in ATP production and in triggering apoptosis [6]. Thus, the consequence of mitochondrial dysfunction in SLE deserves further appraisal. Perl *et al.* [25] reported that mitochondrial dysfunction with ATP depletion that elicit necrosis in the T cells of SLE patients. Decreased copy number of mtDNA, mitochondrial dysfunction and the resulting apoptosis or necrosis in immunocompetent cells might help sustain the vicious cycle and further worsen the SLE course.

Due to the homoplasmic nature of mtDNA, the existence of D310 heteroplasmy may result in mtDNA instability. Because D-loop is the regulatory region for mtDNA replication and D310 is close to the replication origin of mtDNA, the heteroplasmic D310 pattern may interfere with the replication of mtDNA and augment the decrease in mtDNA copy number [13,25]. Taken together, alterations in mtDNA, either the mtDNA D310 sequence variations or fluctuations in mtDNA copy number, might be a good biomarker to reflect the progression of SLE. Although it is difficult to investigate the time-dependent heteroplasmic change of mtDNA as the disease progresses in SLE patients, the results in the present investigation suggest that mtDNA damage may be parallel to disease flares or indicates an active disease status.

In the gene expression study, we found that the mRNA transcripts of the mtDNA-encoded ND1 and ATPase 6 genes in the leukocytes of controls were significantly higher than those of the SLE patients (Table 1) and had a positive correlation with mtDNA copy numbers. However, we observed an increase in leukocyte mtDNA copy number in SLE patients with low SLEDAI as compared to the controls, though this trend was reversed in patients with high SLEDAI (Table 2 and Figure 2A). This paradoxical biphasic alteration is difficult to interpret but it is possible that a feed-back up-regulation of mtDNA replication may take place to compensate for the oxidative damage on mtDNA during the progression of the disease [26–28]. Once it goes too far beyond the capacity of compensation, a decrease in mtDNA copy number follows. This bizarre phenomenon of mtDNA copy number alteration has also been reported in the human lung tissues of light smokers relative to non-smokers and heavy smokers as well as in cell lines challenged by different concentrations of H_2_O_2_ [27,28]. Recently, it was pointed out that an increase in the copy number of circulating mtDNA, which is assumed to be released by the leukocytes bearing dysfunctional mitochondria, is related to a poor outcome in SLE patients [9].

### 2.4. Correlation between D310 Heteroplasmy and Clinical Manifestations of SLE

We used the number of D310 variants to represent the degree of D310 heteroplasmy in leukocyte mtDNA of the patients. As shown in Tables 1 and 2, there was a higher frequency of D310 heteroplasmy in SLE patients with a higher SLEDAI score. To verify the significance of D310 heteroplasmy in SLE, we analyzed the degree of D310 heteroplasmy and its relationship to the clinical manifestations of SLE. As shown in Table 3, the frequency of D310 heteroplasmy did not differ among patients with or without CNS involvement, skin rash, alopecia, oral ulcer or decreased complement. However, patients with lupus nephritis had more D310 variants than those without lupus nephritis (2.5 *vs.* 2.0, *p* = 0.035, Student *t* test).

### 2.5. D310 Sequence Variation and Susceptibility to Clinical Manifestations of SLE

The association between D310 sequence variations and the susceptibility to various clinical manifestations, including CNS involvement, nephritis, skin rash, alopecia, oral ulcer and decreased complement are listed in Table 4. Compared to those harboring other dominant D310 variants, SLE patients with C_9_TC_6_ as the dominant variant had a higher tendency to develop lupus nephritis (52.4% for C_9_TC_6_, 33.3% for C_7_TC_6_ and C_8_TC_6_, *p* = 0.084, Chi-square test).

It is noteworthy that the D310 polymorphism was related to lupus nephritis, which is the most important complication among the lupus related organ injuries [29,30]. The degree of D310 heteroplasmy in nephritis patients was higher than those without nephritis, but not higher than those with other clinical manifestations (Table 3). Most importantly, we demonstrated that the frequency of occurrence of C_9_TC_6_ as the dominant variant in SLE patients was higher than that of controls (Table 1). Reciprocally, SLE patients harboring C_9_TC_6_ as the dominant variant tended to develop nephritis more frequently than patients harboring mtDNA with other D310 variants (Table 4). Therefore, the D310 heteroplasmy of mtDNA is not only a good biomarker to reflect the progression of SLE but also predicts the susceptibility to lupus nephritis, especially in those harboring more D310 variants.

## 3. Experimental Section

### 3.1. Patient Recruitment, Blood Sample Collection, and Leukocyte DNA and RNA Extraction

According to the American College of Rheumatology criteria for the classification of SLE [31,32], a total of 85 SLE patients (73 females) with a mean age of 44.6 ± 12.0 years from the Outpatient Clinic of the Division of Allergy, Immunology and Rheumatology, Taipei Veterans General Hospital and 45 matched healthy controls (38 females) with a mean age of 42.6 ± 9.0 years were recruited for this study. Their demographic data, including gender, age, organ involvement and laboratory profiles, were recorded in detail. Disease activity was evaluated by the SLE Disease Activity Index (SLEDAI) scoring system [33,34]. Approval from the Institutional Review Board of Taipei Veterans General Hospital had been obtained before this study was conducted.

Approximately 10 mL of the venous blood was drawn and kept in specific tubes (VACUETTE^®^, Greiner Bio-one) rinsed with ethylenediaminetetraacetic acid (EDTA). After centrifugation at 3000 *g* for 10 min at 4 °C, the leukocyte-enriched buffy coat was obtained. Following lysis of erythrocytes with 0.83N (NH_4_)_2_SO_4_, the leukocytes were mixed with the TE buffer (10 mM Tris-HCl and 1 mM EDTA, pH 8.0) containing 10% sodium dodecyl sulfate (SDS) (TE:SDS = 10:1) and proteinase K (20 mg/mL) and incubated at 56 °C for 16 h. The leukocyte DNA was then purified through standard phenol-chloroform extraction and isopropanol precipitation procedure as described previously [35]. The DNA samples were dissolved in nuclease-free distilled water and kept at −20 °C until use.

Among the 85 SLE patients and 45 healthy controls, leukocytes from 44 patients and 26 controls were also randomly selected for RNA extraction. As described previously [36], the leukocytes were mixed with 500 μL of TRI^TM^ reagent (Sigma-Aldrich Chemical Co.) and 100 μL of chloroform to centrifuge at 12,000 *g* at 4 °C for 15 min. The supernatant containing RNA was precipitated by 250 μL of isopropanol and centrifuged at 12,000 *g* at 4 °C for additional 10 min to get the RNA pellet. After rinse with 75% alcohol for 2 times, the RNA pellet was kept dry at 4 °C for 30 min and dissolved in distilled water containing 0.1% diethylpyrocarbonate (DEPC). A total of 5 μg RNA was further purified in a reaction buffer containing DNase to remove the residual DNA. Finally, a total of 2 μg of purified DNA-free RNA was reverse-transcribed to cDNA with the Ready-To-Go RT-PCR kit (GE Healthcare UK) by using oligo-dT primers.

### 3.2. Sequencing of the D310 Region of mtDNA

The D310 region of mtDNA was amplified by polymerase chain reaction (PCR) and then subjected to direct sequencing [21]. Each 50-μL PCR reaction contained 25 μL of RBC SensiZyme^®^ Hotstart Taq Premix (RBC Bioscience), 22 μL of PCR-grade H_2_O, 1 μL of each primer (H76-1: 5′-CACGCGATAGCATTGCGA-3′ and L335: 5′-TAAGTGCTGTGGCCAGAAGC-3′), and 1 μL of sample DNA (10 ng/μL). The PCR procedures included a hot start at 95 °C for 10 min, 40 cycles of 95 °C, 15 s; 58 °C, 15 s; and 72 °C, 30 s; and final extension at 72 °C for 7 min. After confirmation by 3% agarose electrophoresis, the PCR products were subjected to direct sequencing (MB Mission Biotech, Taipei, Taiwan). The D310 sequence variations, including the patterns of homoplasmy or heteroplasmy, number of D310 variants, and the dominant D310 variant were compared to the revised Cambridge Reference Sequence’s (rCRS), L strand sequence (MITOMAP; A human mitochondrial genome database) [10,11], and determined as previously described [21,22].

### 3.3. Standard Curves for DNA and RNA Quantification

Quantitative real-time PCR (Q-PCR) using SYBR Green I (Roche Applied Science, Mannheim, Germany) to determine the threshold cycle (Ct) was applied for quantification of DNA and mRNA [37]. For DNA, genomic DNA at different concentrations from the 143B osteosarcoma cells, which was serially diluted by 4-fold from 320 to 0.078125 ng/μL, were subjected to Q-PCR for determination of the Ct values. The sequences of primers used for amplification of mtDNA (ND1 region) and nDNA (18S rRNA region) were mtF3212: 5′-CACCCAAGAACAGGGTTTGT-3′ and mtR3319: 5′-TGGCCATGGGATTGTTGTTAA-3′ and 18SF1546: 5′-TAGAGGGACAAGTGGCGTTC-3′ and 18SR1650: 5′-CGCTGAGCCAGTCAGTGT-3′, respectively [21]. The squared regression coefficient (*R^2^*) for mtDNA was 0.9995, and that for nDNA was 0.9996. For quantification of mRNA transcripts, the cDNA transcribed from 2 μg of purified DNA-free RNA of the 143B osteosarcoma cells was serially diluted by 4-fold from 0.25 (1/4) fold to 0.0009765 (1/1024) and then subjected to Q-PCR for determination of the Ct value. The sequences of primers used for quantification of mtDNA-encoded ND1 (reflecting electron transport) and ATPase 6 (reflecting oxidative phosphorylation) mRNA, and nDNA encoded β-actin mRNA (positive control) were ND1F: 5′-TGGGTACAATGAGGAGTAGG-3′and ND1R: 5′-GGAGTAATCCAGGTCGGT-3′; and ATPase 6F: 5′-TTTATTGCCACAACTAACCTCCT-3′ and ATPase 6R: 5′-TTGGGTGGTTGGTGTAAATG-3′ and BAF: 5′-ATTGGCAATGAGCGGTTC-3′ and BAR: 5′-GGATGCCACAGGACTCCAT-3′, respectively. The squared regression coefficients (*R^2^*) were 0.9999 for ND1, 0.9997for ATPase 6, and 0.9985 for β-actin, respectively [38,39].

### 3.4. Determination of mtDNA Copy Number and the Expression of mtDNA-Encoded Genes

The mtDNA copy number was defined as total mtDNA copies divided by total nDNA copies. The mtDNA-encoded mRNA expression was defined as total ND1 or ATPase 6 copies divided by total β-actin copies. For each reaction, 1 μL of sample DNA (10 ng/μL)/1 μL of cDNA (16 × dilution) was amplified in a 10-μL reaction buffer containing 0.25 μL (20 μM) of each primer (mtF and mtR for mtDNA, 18SF and 18SR for nDNA; ND1F and ND1R and ATPase 6F and ATPase 6R for mtDNA-encoded mRNA, BAF and BAR for nDNA-encoded mRNA), 1.2 μL of 3 mM MgCl_2_, 1 μL of LightCycler SYBR Green I mixed reagent (Roche Applied Science, Mannheim, Germany) and 6.3 μL of PCR grade H_2_O. Simultaneously, 1 μL of DNA (1 ng/μL)/cDNA (16 × dilution) from 143B cells and PCR grade H_2_O were included as the positive and negative controls, respectively. The PCR procedures included a hot start at 95 °C, 10 min and 40 cycles of 95 °C, 20 s; 62 °C, 20 s; and 72 °C, 20 s. The fluorescence intensity was measured at the end of primer extension at 72 °C for Ct calculation. The mtDNA copy number (total mtDNA copies/total nDNA copies) and mRNA levels of mtDNA-encoded ND1 or ATPase 6 genes (total ND1 copies/total β-actin copies or total ATPase 6 copies/total β-actin copies) of each clinical sample was calculated, by adjusting the 143B cell mtDNA copy number and the mRNA level of mtDNA-encoded genes as 1. Each reaction was done in duplicate and the mean value was used for data presentation [21].

### 3.5. Statistical Analysis

All the statistical analyses were performed using Statistical Package for the Social Sciences (SPSS), version 15.0, software (SPSS Inc., Chicago, IL, USA, 2006). The continuous variables were compared using the Student’s *t* test/Mann-Whitney *U* test between two groups or ANOVA/Kruskal-Wallis *H* test among three or more groups when appropriate. Categorical variables between groups were compared using the Chi-square test/Fisher’s exact test or Chi-square test for trend when appropriate. The difference between groups was considered significant when the *p* value was less than 0.05.

## 4. Conclusions

In conclusion, the results in this study showed a decrease in mtDNA copy number and greater heteroplasmic change in the D310 region of mtDNA in leukocytes of SLE patients. We suggest that leukocyte mtDNA alterations might be relevant to the development and progression of SLE. Furthermore, the SLE patients harboring higher D310 heteroplasmy in leukocytes may be more susceptible to lupus nephritis.

## Figures and Tables

**Table 1:** Demographic data and mtDNA characteristics of normal controls and systemic lupus erythematosus (SLE) patients.

Demographic data/mtDNA characteristics	Controls	SLE patients	*p*-value
**Case number (%)**	45 (100.0)	85 (100.0)	-
**Gender**	-	-	0.825 ^*^
Male (**%**)	7 (15.6)	12 (14.1)	-
Female (**%**)	38 (84.4)	73 (85.9)	-
**Age (mean ± SD) years**	42.6 ± 9.0	44.6 ± 12.0	0.335 ^§^
**SLEDAI**	-	-	-
Min~Max, Median	-	2~28, 8	-
**Mitochondrial DNA**			
D310 sequence variation, predominant variant	-	-	0.079 ^*^
C_5_TC_6_ (%)	1 (2.2)	0 (0.0)	-
C_7_TC_6_ (%)	19 (42.2)	27 (31.8)	-
C_8_TC_6_ (%)	18 (40.0)	36 (42.4)	-
C_9_TC_6_ (%)	5 (11.1)	21 (24.7)	-
C_10_TC_6_ (%)	2 (4.4)	0 (0.0)	-
C_11_TC_6_ (%)	0 (0.0)	1 (1.2)	-
D310 patterns	-	-	0.022 ^*^
Heteroplasmy (%)	22 (48.9)	59 (69.4)	-
Homoplasmy (%)	23 (51.1)	26 (30.6)	-
Number of D310 variants (mean ± SD)	1.7 ± 0.8	2.2 ± 1.1	0.014 ^§^
mtDNA copy number (mean ± SD)	0.193 ± 0.065	0.214 ± 0.113	0.169 ^§^
**Case number (%)** †	26 (100.0)	44 (100.0)	-
**mtDNA encoded mRNA expression**			
ND1 (mean ± SD)	2.845 ± 2.000	1.625 ± 1.029	0.001 ^§^
ATPase 6 (mean ± SD)	1.538 ± 1.108	0.928 ± 0.526	0.003 ^§^

*p* value was calculated by ^*^ Chi-square test, or ^§^ Student *t* test. ND1 = NADH dehydrogenase subunit 1. ATPase 6 = ATP synthase subunit 6.

† Randomly selected from original patients and controls.

**Table 2:** Correlation between SLEDAI scores and mtDNA alterations in SLE patients.

	Control	SLE			*p-*value

		SLEDAI ≤ 4.0@	4.0@ < SLEDAI ≤ 14.0@	SLEDAI > 14.0@	
**Case number (%)**	45 (100.0)	26 (100.0)	42 (100.0)	17 (100.0)	-
**Mitochondrial DNA**					
D310 pattern	-	-	-	-	0.021 *
Heteroplasmy (%)	22 (48.9)	17 (65.4)	29 (69.0)	13 (76.5)	-
Homoplasmy (%)	23 (51.1)	9 (34.6)	13 (31.0)	4 (23.5)	-
mtDNA copy number (mean ± SD)	0.193 ± 0.065	0.243 ± 0.147	0.211 ± 0.098	0.179 ± 0.077	0.119 **
	0.193 ± 0.065	0.243 ± 0.147	-	-	0.049 a
	-	0.243 ± 0.147	-	0.179 ± 0.077	0.068 b

@ 4.0 was at 25 percentile and 14.0 was at 75 percentile.

* Comparison was made among the 4 groups by Chi-square test for trend.

** Comparison was made among the 4 groups by ANOVA.

a Compared between controls and those with SLEDAI ≤ 4.0 by Student *t* test.

b Compared between those with SLEDAI ≤ 4.0 and those with SLEDAI > 14.0 by Student *t* test.

**Table 3:** D310 heteroplasmy in SLE patients with different clinical manifestations.

	D310 pattern			Number of D310 variants	
				
Case number	Heteroplasmic No. (%)	Homoplasmic No. (%)	*p*-value *	(Mean ± SD)	*p*-value **
CNS involvement					
No (*n* = 42) (100%)	29 (69.0)	13 (31.0)	0.943	2.1 ± 1.0	0.860
Yes (*n* = 43) (100%)	30 (69.8)	13 (30.2)		2.2 ± 1.2	
Nephritis					
No (*n* = 52) (100%)	35 (67.3)	17 (32.7)	0.597	2.0 ± 0.9	0.035
Yes (*n* = 33) (100%)	24 (72.7)	9 (27.3)		2.5 ± 1.3	
Skin rash					
No (*n* = 65) (100%)	45 (69.2)	20 (30.8)	0.948	2.2 ± 1.1	0.604
Yes (*n* = 20) (100%)	14 (70.0)	6 (30.0)		2.1 ± 1.1	
Alopecia					
No (*n* = 59) (100%)	39 (66.1)	20 (33.9)	0.318	2.1 ± 1.1	0.325
Yes (*n* = 26) (100%)	20 (76.9)	6 (23.1)		2.3 ± 1.2	
Oral ulcer					
No (*n* = 72) (100%)	52 (72.2)	20 (27.8)	0.186	2.2 ± 1.1	0.268
Yes (*n* = 13) (100%)	7 (53.8)	6 (46.2)		1.8 ± 1.1	
Decreased complement					
No (*n* = 54) (100%)	36 (66.7)	18 (33.3)	0.469	2.2 ± 1.2	0.413
Yes *** (*n* = 31) (100%)	23 (74.2)	8 (25.8)		2.0 ± 0.9	

* Calculated by Chi-square test.

** Calculated by Student *t* test.

*** Decreased complement means serum complement components C3 and C4 were both below the cutoff level of the normal range.

**Table 4:** Association of the D310 sequence variations of mtDNA with different clinical manifestations in SLE patients.

	D310 sequence variation, predominant variant (Case number, %)				
	
	C_7_TC_6_	C_8_TC_6_	C_9_TC_6_	C_12_TC_6_	
	*n* = 27 (100)	*n* = 36 (100)	*n* = 21 (100)	*n* = 1 (100)	*p*-value *
CNS involvement					
No (*n* = 42)	14 (51.9)	16 (44.4)	12 (57.1)	0 (0.0)	0.594
Yes (*n* = 43)	13 (48.1)	20 (55.6)	9 (42.9)	1 (100.0)	
Nephritis					
No (*n* = 52)	18 (66.7)	24 (66.7)	10 (47.6)	0 (0.0)	0.084
Yes (*n* = 33)	9 (33.3)	12 (33.3)	11 (52.4)	1 (100.0)	
Skin rash					
No (*n* = 65)	21 (77.8)	24 (66.7)	19 (90.5)	1 (100.0)	0.208
Yes (*n* = 20)	6 (22.2)	12 (33.3)	2 (9.5)	0 (0.0)	
Alopecia					
No (*n* = 59)	22 (81.5)	22 (61.1)	14 (66.7)	1 (100.0)	0.316
Yes (*n* = 26)	5 (18.5)	14 (38.9)	7 (33.3)	0 (0.0)	
Oral ulcer					
No (*n* = 72)	21 (77.8)	31 (86.1)	19 (90.5)	1 (100.0)	0.620
Yes (*n* = 13)	6 (22.2)	5 (13.9)	2 (9.5)	0 (0.0)	
Decreased complement					
No (*n* = 54)	19 (70.4)	19 (52.8)	15 (71.4)	1 (100.0)	0.323
Yes (*n* = 31)	8 (29.6)	17 (47.2)	6 (28.6)	0 (0.0)	

* Calculated by Chi-square test.
